# Complete genome sequence of *Vibrio parahaemolyticus* ST36 strain MAVP-26, a clinical isolate from an oyster-borne human gastric infection

**DOI:** 10.1128/mra.00352-24

**Published:** 2024-06-12

**Authors:** Randi L. Foxall, Feng Xu, Robert P. Sebra, Vaughn S. Cooper, Stephen H. Jones, Cheryl A. Whistler

**Affiliations:** 1 Northeast Center for Vibrio Disease and Ecology, University of New Hampshire, Durham, New Hampshire, USA; 2 Department of Molecular, Cellular, & Biomedical Sciences, University of New Hampshire, Durham, New Hampshire, USA; 3 Ichan School of Medicine at Mt Sinai, New York, New York, USA; 4 Department of Microbiology and Molecular Genetics, University of Pittsburgh School of Medicine, Pittsburgh, Pennsylvania, USA; 5 Department of Natural Resources and the Environment, University of New Hampshire, Durham, New Hampshire, USA; Department of Biology, Queens College, Queens, New York, USA

**Keywords:** *Vibrio parahaemolyticus*, oyster, gastroenteritis, pathogenicity islands, inovirus

## Abstract

A Pacific native lineage of *Vibrio parahaemolyticus* ST36 serotype O4:K12 was introduced into the Atlantic, which increased local source illnesses. To identify genetic determinants of virulence and ecological resiliency and track their transfer into endemic populations, we constructed a complete genome of a 2013 Atlantic-traced clinical isolate by hybrid assembly.

## ANNOUNCEMENT

The introduction of Pacific native *V. parahaemolyticus* into the North Atlantic has raised concerns about how the non-native *V. parahaemolyticus* may evolve under local selection or even spur the evolution of new variants by recombination with endemic lineages (1–6). This is especially concerning considering that Vibrio pathogenicity islands (VPaI), which confer disease in humans (7), are mobile by nature. Therefore, we sequenced reference strain MAVP-26, a 2013 clinical isolate (8, 9). The vector of the gastric illness was a commercial oyster definitively traced to a harvest area in Cape Cod Bay (42.0201° N; 70.6514° W), where the lineage is now established (2).

Using standard microbial methods for pathogenic *Vibrio* (10), the isolate was cultured from a stool sample on thiosulfate citrate bile salt sucrose agar (TCBS) and cryopreserved by the Massachusetts Department of Public Health. Genomic DNA was isolated from cells grown in Luria–Bertani (LB) broth at 37° with aeration, using the alkaline lysis/detergent protocol, followed by organic extraction, a method that produces high-molecular weight DNA (11), and sequenced using the Pacific Biosciences RSII technology. Library preparation and sequencing were carried out as per the manufacturer’s instructions (Pacific Biosciences, Menlo Park CA, USA) and reflects the P6-C4 enzyme and chemistry, as previously described (1). Briefly, DNA was sheared to ~20,000 bp, purified, and repaired. SMRTbell adapters were end-ligated, and un-ligated fragments were enzymatically removed. Following size-selection to 7,000 bp–50,000 bp, the primer was annealed, the polymerase–template complex was bound and loaded onto magnetic beads, and then placed onto the RSII machine configured for 180-minute continuous sequencing.

The genome was assembled *de novo* from a total of 124,153,197 long reads (average length of 18,444 bp, N50 = 31,941) using CANU1.6 (12) which corrects, trims, and assembles the reads. Subsequently, overhangs were identified on chromosomes 1 and 2 and trimmed, and each chromosome circularized using Circlator 1.5.5 (13). Chromosome 1 was oriented so that the start position was at *dnaA*. Adapter-trimmed (14) Illumina HiSeq 2500 150-bp, paired-end reads [(average read length of 151 bp, 249 x coverage, SRA: SRS893228 (1)] were aligned to the PacBio assembly using BWA (15), indexed using samtools (16), and errors (including relatively few single-nucleotide polymorphisms and small indels) corrected using Pilon version 1.20 (17) with a final genome assembly coverage of 56 x. Annotations were produced upon submission by NCBI Prokaryotic Genome Annotation Pipeline (PGAP) v.4.2 using the best-placed reference protein set and GeneMarkS+ (18, 19) and deposited in the NCBI RefSeq database (20). Default parameters were used for all software programs used for the publicly available genome. The research does not involve interaction or intervention with human subjects and the researchers did not have access to identifiable private information.

**Fig 1:**
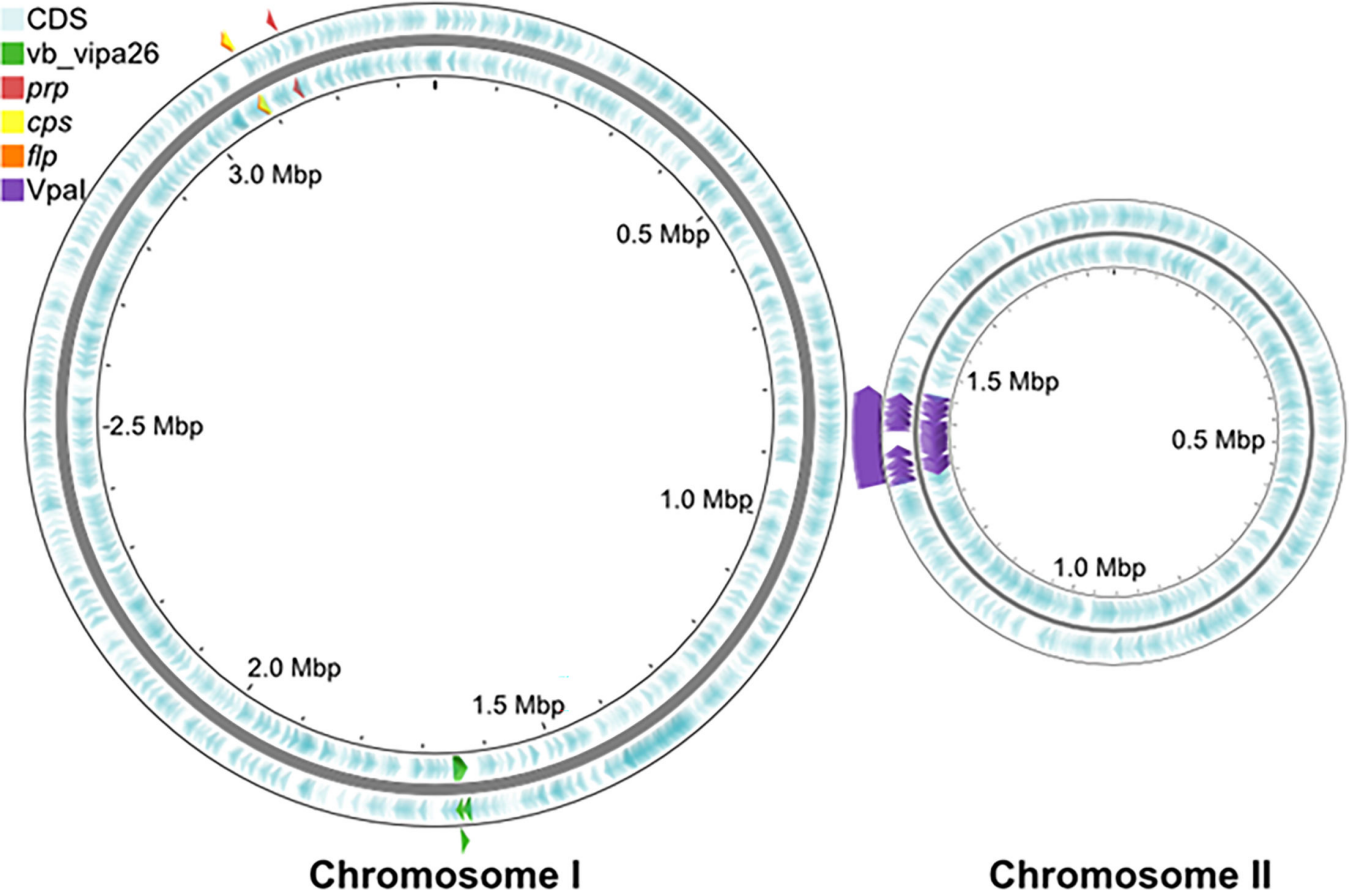
Depiction of two chromosomes of MAVP-26 with notable diagnostic features and mobile elements. Coding sequences are shown in light blue, and key genomic features and positions are shown. Inovirus phage vb_vipa26 that is diagnostic of this population is in green, marker *prp* in red, marker *cps* in yellow, marker *flp* in orange, and the pathogenicity island VPaIγ harboring hemolysin genes *tdh* and *trh* in purple. Visually produced with proksee software (https://proksee.ca).

The genome contains two circular chromosomes, chromosome I (3,360,050 bp) and chromosome II (1.849,390 bp), encoding 5,047 genes with 45.5% GC content. Notable mobile elements including the pathogenicity island (VPaI-γ) and inovirus (1, 2) and other diagnostic features (9) are indicated (Fig. 1).

## Data Availability

This Whole Genome Shotgun project has been deposited in GenBank under the accession number CP023248 (chromosome I) and CP023247 (Chromosome II). The PacBio raw reads are available under the accession number SRR28518279, and the Illumina short reads are available under the accession number SRS893228 (2). This is the second version of the complete hybrid assembly genome of *V. parahaemolyticus* MAVP-26, and in addition to the previous hybrid assembly, a prior assembly (30 contigs, N50 = 809,100) from the Illumina reads was previously released under the RefSeq accession number GCF_001023055.1 and GenBank WGS is LBHD00000000.1 (2).
